# Characteristics of youth who leave the emergency department without being seen following sports-related concussion

**DOI:** 10.2217/cnc-2019-0010

**Published:** 2019-12-06

**Authors:** Jacquelyn J Deichman, Janessa M Graves, Tracy A Klein, Jessica L Mackelprang

**Affiliations:** 1 College of Nursing, Washington State University, Spokane, WA 99210, USA; 2 Honors College, Washington State University, Pullman, WA 99164, USA; 3 College of Nursing, Washington State University, Vancouver, WA 98686, USA; 4 School of Health Sciences, Swinburne University of Technology, Melbourne, Victoria, 3122, Australia

**Keywords:** adolescents, disparities, emergency medicine, equity, race, youth

## Abstract

**Aim:**

Despite the rising incidence of emergency department (ED) visits for sports-related concussion, the frequency and characteristics of youth leaving before being seen are unknown.

**Methodology:**

National estimates of ED visits for sports-related head injuries among youth (10–18 years) were generated for 2006–2017 using the National Electronic Injury Surveillance System. Logistic regression models estimated the odds of leaving without being seen across patient characteristics and time.

**Results:**

From 2006 to 2017, 985,966 (95% CI: 787,296–1,184,637) ED visits were identified for sports-related concussions, of which 5015 (95% CI: 3024–7006) left without being seen.

**Conclusion:**

Youth with sports-related concussion must receive timely care and ED improvements may reduce rates of leaving without being seen.

## Lay abstract

Over the last decade, more and more youth have visited the emergency department (ED) for sports-related concussion. At the same time, EDs are becoming more crowded, with long wait times and many patients leaving before being evaluated. An increase in the proportion of youth with concussion who left without being seen (LWBS) is possible. We used national data of ED visits to examine the rate of youth (aged 10–18 years) who LWBS for sports-related concussion from 2006 to 2017. While the rate of LWBS has not changed over time, more nonwhite youth and youth who were not injured at school left before being evaluated.

Each year in the USA, an estimated 1.1–1.9 million youth experience sports- and recreation-related concussions [1]. To address growing concerns about concussions and the potential consequences of repeat injuries and secondary concussion syndrome [2], return-to-play legislation has been adopted in all states and the District of Columbia. Policies vary by state, but all generally restrict return-to-sports eligibility until evaluation and consent to return are obtained from a healthcare provider [3]. Among youth with sports-related concussions, an estimated 33–54% are treated by healthcare providers annually, the majority of whom are seen in outpatient or emergency department (ED) settings [1,4]. Among 10- to 18-year-old youth, an estimated 191,141 sports and recreation-related traumatic brain injuries were seen in EDs in 2016 [5]. Over time, US EDs have seen an increase in the number of youth presenting with concussions [6,7].

Overcrowding is prevalent in EDs across the USA [8,9]. Long wait times and ED congestion result in increased rates of patients leaving without being evaluated by a healthcare provider, termed ‘left without being seen’ (LWBS) [9]. This phenomenon contributes to service inefficiency, poor health outcomes and increased healthcare costs [10]. To date, no study has examined LWBS among youth seeking ED care for concussion.

Return-to-play legislation and heightened concussion awareness have contributed to an increase in ED visits for suspected concussions (including among lower acuity injuries) [7,11,12]. Whether the incidence of LWBS has increased is unknown. The purpose of this study was to examine characteristics of youth who LWBS from the ED following sports-related concussion.

## Methods

This retrospective, cross-sectional study used data from the National Electronic Injury Surveillance System (NEISS), a national probability sample of 100 US hospitals’ emergency departments (EDs), each reporting information associated with product-related injuries [13]. The NEISS is administered by the US Consumer Product Safety Commission (MD, USA) and serves as a public health monitoring tool to examine ongoing, emerging and changing trends in ED-reported injuries related to consumer products. Injury and visit information are extracted from ED visit data and recorded by trained coders at NEISS hospitals. National estimates of product-related injuries are generated using NEISS data.

Subjects included were youth, aged 10–18 years, who were presented to EDs with sports-related head injury between 1 January 2006 to 31 December 2017. In this age range (middle and high school), most youth report involvement in at least one organized sport [14,15], and organized sports are frequently offered through schools, starting in middle school. Cases were identified as those involving sports-related equipment for the following team sports: football, basketball, soccer, baseball, softball, lacrosse, ice hockey, cheerleading and volleyball. Concussion injuries were identified as cases with a head injury (body part code 75) and diagnosis of a concussion or internal organ injury (diagnosis codes 52 or 62; consistent with previous studies [16]). Cases that resulted in LWBS were identified using the patient disposition variable reported in NEISS (‘left without being seen/left against medical advice’).

Return-to-play legislation applies to youth involved in school sports; the sample was, therefore, restricted to injuries occurring at school or a place of recreation. Injuries coded as the latter were included because many schools train or host sport competitions outside school property. Patient demographic characteristics included sex (male, female), age (10–12, 13–15 and 16–18 years) and race (white, nonwhite and unknown). The NEISS database does not report detailed information on hospital or patient characteristics, such as location, insurance status, International Classification of Diseases (ICD)-10 diagnosis codes, procedures, time of visit or mode of arrival.

To examine the potential association between LWBS and time, three time periods were designated based on national adoption of return-to-play legislation. The time period before any US state passed return-to-play legislation (‘prelegislation’) occurred from 1 January 2006 to 31 December 2008. The interim time period spanned 1 January 2009–31 December 2014. The ‘postlegislation’ time period, after every state had adopted return-to-play legislation, was from 1 January 2015 to 31 December 2017.

Statistical analyses were conducted using Stata/MP version 14 (Stata, TX, USA), using svy commands to account for survey design. National estimates of injuries and 95% CIs were calculated using NEISS sample weights. Demographic and injury characteristics were summarized across cases. The proportion of ED visits that LWBS was calculated as the number of cases with discharge disposition coded as LWBS, divided by total number of concussion-related ED visits in each time period.

Bivariate relationships between factors (patient demographics, injury characteristics and time [coded as year or time period]) and discharge status (LWBS vs not) were evaluated using Pearson's design-based chi-squared tests. Multivariable logistic regression models estimated the odds of LWBS for each factor while controlling for co-variates. Results were presented as odds ratios with 95% CIs, demonstrating the relative odds of LWBS, given each co-variate of interest (e.g., race). Time period was included in models as a categorical variable in order to identify potential variation in LWBS over time. As a sensitivity analysis, we created linear spline time functions for each time period (using the Stata function mkspline) and included these in the model (in lieu of the categorical time variable) in order to identify potential variation in LWBS across time periods. This study was deemed exempt from human subjects review by the Washington State University Institutional Review Board due to the de-identified nature and public availability of the study data.

## Results

From 1 January 2006 to 31 December 2017, 985,966 (95% CI: 787,296–1,184,637) ED visits among youth aged 10–18 years with sports-related head injuries were identified. Most were males (70.8%) whose injuries occurred at recreational locations (away from school) and were associated with football, basketball and soccer (Table 1).

**Table 1.** Characteristics and discharge disposition of youth presented to emergency departments with sports-related concussions, 2006–2017.

Variable	Cases seen (n = 980,952)	Cases left without being seen (n = 5015)	Total (n = 985,966)	Significance
**Demographic characteristics**				
Sex				0.56
– Male	70.8	66.9	70.8	
– Female	29.2	33.1	29.2	
Age (years)				0.10
– 10–12	23.2	32.0	23.2	
– 13–15	44.6	36.8	44.5	
– 16–18	32.3	31.3	32.3	
Race				<0.01
– White	53.1	32.3	53.0	
– Non-white	18.5	34.8	18.6	
– Unknown/missing	28.5	33.0	28.5	
**Injury characteristics**				
Sport type				0.65
– Football	39.3	35.0	39.3	
– Basketball	20.0	19.8	20.0	
– Soccer	16.7	19.8	16.8	
– Baseball	7.6	8.6	7.6	
– Softball	4.4	5.0	4.4	
– Cheerleading	3.8	4.4	3.8	
– Ice hockey	3.0	1.0	3.0	
– Lacrosse	2.6	1.5	2.6	
– Volleyball	2.7	4.9	2.7	
Diagnosis				<0.01
– Concussion	52.1	9.2	51.9	
– Internal organ injury	47.9	90.1	48.1	
Injury locale				<0.01
– School	33.1	22.0	33.0	
– Place of recreation	66.9	78.0	67.0	

Based on national estimates. Values indicate percent of column totals. Percentages may not sum up to 100 due to rounding. Significance based on p-value from Pearson's design-based chi-squared test.

In total, 5015 (95% CI: 3024–7006) ED visits resulted in LWBS, representing an estimated 0.51% (95% CI: 0.38–0.68) of all cases. Cases that resulted in LWBS were more frequently nonwhite or unknown race (versus white), had a diagnosis of internal organ injury to the head (versus concussion) and occurred at a place of recreation rather than at school (p < 0.01; Table 1). In the 3 years before return-to-play laws were implemented nationally, 630 (95% CI: 149–1111) visits resulted in LWBS, compared with 1665 (95% CI: 841–2490) in the 3 years after all states adopted return-to-play laws. Proportionally, this equates to 0.41% (95% CI: 0.21–0.81) and 0.57% (95% CI: 0.38–0.85) of visits LWBS before and after return-to-play law adoptions, respectively; this change was not statistically significant (p = 0.66). *Post hoc* power calculation showed sufficient power (100%) to detect this difference in LWBS across time periods.

Associations identified in multivariable logistic regression models were consistent with chi-squared tests. Multivariable models did not show an association between the incidence of LWBS over time, either yearly or by prelegislation, interim or postlegislation time periods (Models 1 and 2, Table 2). Compared with youth who were white, youth of nonwhite or unknown race had significantly greater odds of LWBS, adjusting for demographics, injury characteristics and year or time period (Table 2). After controlling for all other factors, patients whose injury was diagnosed as an internal organ injury had significantly greater odds of LWBS, and injuries coded as occurring at school had 45% lower odds of LWBS, after accounting for demographic, injury and time factors (Table 2). The sensitivity analysis using splines for time periods generated similar findings (results not shown).

**Table 2.** Factors associated with youth leaving the emergency department without being seen for sports-related concussion, 2006-2017.

Variable	Unadjusted models	Model 1^‡^	Model 2^§^
	OR (95% CI)	OR (95% CI)	OR (95% CI)
**Demographic characteristics**			
Sex			
– Male	Ref.	Ref.	Ref.
– Female	1.20 (0.67–2.17)	1.20 (0.63–2.30)	1.19 (0.63–2.24)
Age (years)			
– 10–12	1.67 (1.09–2.56)	1.49 (0.93–2.41)	1.48 (0.92–2.37)
– 13–15	1.17 (0.68–2.03)	1.18 (0.68–2.06)	1.20 (0.70–2.05)
– 16–18	Ref.	Ref.	Ref.
Race			
– White	Ref.	Ref.	Ref.
– Nonwhite	3.10 (2.01–4.77)	2.87 (1.79–4.59)	2.84 (1.77–4.58)
– Unknown	1.90 (1.12–3.25)	1.78 (1.02–3.11)	1.74 (0.99–3.06)
**Injury characteristics**			
Sport type			
– Football	Ref.	Ref.	Ref.
– Basketball	1.11 (0.60–2.06)	0.85 (0.46–1.57)	0.86 (0.46–1.58)
– Soccer	1.33 (0.74–2.40)	1.09 (0.60–1.97)	1.08 (0.59–1.98)
– Baseball	1.28 (0.55–3.00)	0.87 (0.35–2.19)	0.87 (0.36–2.13)
– Softball	1.27 (0.49–3.30)	0.89 (0.38–2.09)	0.91 (0.39–2.16)
– Cheerleading	1.29 (0.40–4.16)	1.04 (0.36–3.04)	1.04 (0.35–3.12)
– Ice hockey	0.38 (0.09–1.64)	0.29 (0.07–1.25)	0.29 (0.07–1.25)
– Lacrosse	0.66 (0.23–1.90)	0.65 (0.22–1.89)	0.63 (0.22–1.84)
– Volleyball	2.03 (0.66–6.30)	1.77 (0.68–4.64)	1.78 (0.69–4.58)
Diagnosis			
– Concussion	Ref.	Ref.	Ref.
– Internal organ injury	10.73 (4.82–23.90)	9.99 (4.53–22.05)	9.91 (4.46–22.03)
Injury locale			
– School	0.57 (0.35–0.93)	0.55 (0.33–0.91)	0.55 (0.32–0.92)
– Place of recreation	Ref.	Ref.	Ref.
**Time**			
Year			
– 2006	Ref.	Ref.	-
– 2007	0.18 (0.04–0.89)	0.17 (0.04–0.81)	-
– 2008	0.68 (0.22–2.15)	0.69 (0.22–2.13)	-
– 2009	0.99 (0.30–3.24)	0.86 (0.26–2.79)	-
– 2010	0.41 (0.13–1.35)	0.34 (0.10–1.16)	-
– 2011	0.72 (0.20–2.67)	0.67 (0.17–2.57)	-
– 2012	0.91 (0.29–2.87)	0.82 (0.25–2.68)	-
– 2013	0.77 (0.19–3.22)	0.71 (0.17–2.96)	-
– 2014	0.72 (0.21–2.49)	0.68 (0.18–2.53)	-
– 2015	0.74 (0.21–2.57)	0.70 (0.20–2.46)	-
– 2016	0.70 (0.19–2.50)	0.63 (0.19–2.16)	-
– 2017	1.11 (0.36–3.44)	1.02 (0.33–3.13)	-
Grouped time period			
– 2006–2008 (prelegislation)	Ref.	-	Ref.
– 2009–2014 (interim)	1.22 (0.54–2.76)	-	1.11 (0.46–2.66)
– 2015–2017 (postlegislation)	1.39 (0.66–2.93)	-	1.30 (0.59–2.88)
– Constant		0.0008	0.0005

Values indicate percent of total population during each time period.

‡ Model 1 adjusts for individual year as a dummy variable, patient demographics and injury characteristics.

§ Model 2 adjusts for grouped time period, patient demographics and injury characteristics.

LWBS: Left without being seen; OR: Odds ratio.

## Discussion

Sports-related concussions occur frequently among US youth, with increasing incidence and reporting over time [11]. The high incidence of concussion contributes to growing healthcare costs [17], lost productivity among parents caring for youth postconcussion [18] and school absenteeism [19]. Youth concussion could also theoretically contribute to school presenteeism, wherein students attend but cannot fully participate in school due to concussion-related symptoms, such as fatigue, light sensitivity or headache. In order to avoid these negative outcomes and potentially prolonged symptoms, it is critical that youth receive quality, timely care for their injury.

Findings from this study show that youth LWBS from the ED infrequently. Only 0.51% of visits resulted in LWBS, a rate consistent with other studies examining other injuries using NEISS data [20–23]. Other ED data sources, such as the National Hospital Ambulatory Medical Care Survey, report higher rates of LWBS (1.8–2.6%, not specifically injury) [24,25]. The lower rate of LWBS among youth with sports-related concussion in this study may be attributed to the nature of the concussion injury versus other conditions seen in the ED. Our study also did not find an increase in the incidence of LWBS since return-to-play laws were adopted nationally, despite increasing youth concussion presentations [26,27]. Still, an estimated 511,590–1,240,972 sports-related concussions are not treated by healthcare providers each year [1], which remains cause for concern.

Youth who leave EDs before receiving treatment may or may not seek care from another venue (e.g., outpatient clinic). Indeed, a study of undergraduate students with history of concussion (mean age at injury: 14 years) reported that a third did not seek care for their injury [27]. Commonly cited reasons for not seeking treatment for concussion include mild symptoms, cost and access challenges (e.g., difficulty scheduling an appointment, transportation) [27,28]. It is important to recognize and identify strategies to ensure patients receive timely concussion care, because delaying or avoiding concussion care may lead to undertreatment of ongoing symptoms, delayed recovery or adverse outcomes.

We found a significant association between LWBS and nonwhite or unknown race, compared with white race. Across regression models, nonwhite youth had two- to three-times higher odds of LWBS than their nonwhite peers (adjusting for demographics, injury and time). This apparent disparity in ED disposition by race is concerning. Previous research has shown a similar association between nonwhite race and LWBS [24,29,30]; however, this study is the first to identify potential racial disparities in LWBS among youth with concussion. Given that racial disparities in ED treatment of concussion exist [31], further research with more detailed data is needed to examine this and other possible factors associated with treatment and disposition among youth with concussion.

Educational efforts can encourage youth with sports-related concussion to receive timely care. Programs, such as the Centers for Disease Control and Prevention's (GA, USA) Head's Up campaign, raise awareness of the importance of timely evaluation and treatment of sports-related concussion. The continuation and expansion of these efforts is necessary, including use of innovative communication and outreach strategies to reach vulnerable populations, such as rural or low-income communities.

This study has several limitations. First, the NEISS was designed for injury surveillance purposes and, therefore, it lacks detailed patient information that would be informative when examining LWBS. For example, several known factors associated with LWBS are not captured by NEISS (e.g., time of day, hospital location, insurance status, mode of arrival and injury acuity) [24]. The coding schemes used in NEISS are unique to their dataset and, while it is considered to generate comparable to estimates based on ICD diagnosis codes [32,33], it lacks the specificity to examine outcomes across more granular injury groups. For example, we were unable to examine LWBS across levels of concussion severity or specific symptoms. The NEISS database also does not provide geographic location, so it was not possible to conduct a formal return-to-play policy evaluation across states or by patient residence. Second, there could be limitations in the accuracy of the data due to inconsistent data entry, especially for LWBS cases for which coders may have limited information, or data entry errors. Third, comprehensive details about youth who LWBS may not have been collected by ED staff due to their premature departure. For example, it is possible that information about race was not adequately captured at the initial ED triage, hence the large number of LWBS cases with unknown race. This would also potentially be the case for other variables, such as location where the injury occurred.

Despite these limitations, this study adds to the literature by providing national estimates on the incidence of LWBS for youth who present for ED care following sport-related concussion, as well as factors associated with LWBS in this population. This information lays the foundation for future research to prevent LWBS among youth seeking ED care for concussion and examine outcomes after LWBS.

## Conclusion

Increasing numbers of youth with sports-related concussion are seeking ED treatment for their injuries. From 2006 to 2017, 5015 youth with sports-related concussion left the ED prior to receiving treatment, representing a small percentage of the overall proportion of youth seeking concussion care at the ED (0.51%). It is important to ensure the treatment needs of youth with sports-related concussion are met. To further understand the impact of LWBS on youth with concussion, future research examining outcomes, including physical health, mental health, academic and social outcomes, among youth who LWBS and those who did not is warranted.

## Future perspective

As Americans increasingly rely on the ED as a primary source of medical care, wait times will continue to rise and crowding will worsen. EDs may not serve the needs of youth who must obtain medical clearance before returning to play following sports-related concussion; instead, they may shift to seek care through primary or urgent care. For minor concussions, this may be an appropriate approach to obtaining timely care. However, for more severe injuries, first seeking care elsewhere may result in delays that could contribute to negative outcomes following concussion.

**Summary points**

- Sports-related concussions are a growing concern in the USA. Increasingly, youth are seeking care in the emergency department following sports-related concussion.
- This study examined emergency department data for youth with sports-related head injury who left the emergency department before receiving treatment to evaluate changes over time and identify characteristics of youth who left compared with those who did not.
- From 2006 to 2017, 5015 youth with sports-related head injury left the emergency department before receiving care.
- Youth identified as nonwhite were significantly more likely to leave the emergency department without being seen, compared with white youth.
- Following sports-related head injury, it is important that all youth receive timely, high-quality care in order to identify potential complications and ensure a safe and speedy recovery.
